# A new species of *Nitocra* Boeck, 1865 (Harpacticoida, Ameiridae, Ameirinae) from South Africa, with notes on its ecology and remarks on the status of *Nitocra sewelli husmanni* Kunz, 1976

**DOI:** 10.3897/zookeys.244.2633

**Published:** 2012-11-20

**Authors:** Samuel Gómez, Nicola K. Carrasco, Francisco Neptalí Morales-Serna

**Affiliations:** 1 Instituto de Ciencias del Mar y Limnología, Unidad Académica Mazatlán; Joel Montes Camarena s/n, 82040, Mazatlán, Sinaloa, México; 2 School of Life Sciences, University of KwaZulu-Natal, Westville Campus, P. Bag. X54001, Durban 4000, South Africa; 3 Departamento de Zoología, Instituto de Biología, Universidad Nacional Autónoma de México, Avenida Universidad 3000; Ciudad Universitaria; C.P. 04510, Mexico, D.F.

**Keywords:** *Nitocra*, taxonomy, ecology, St Lucia Estuary, South Africa

## Abstract

A new species of the genus *Nitocra* Boeck, 1865, *Nitocra taylori* **sp. n.** is described from the St Lucia Estuary, Africa’s largest estuarine lake. It is also suggested that *Nitocra sewelli husmanni* Kunz, 1976 and *Nitocra reducta fluviatilis*Galhano, 1968 are granted full species rank as *Nitocra husmanni* **stat. n.** Kunz, 1976 and *Nitocra fluviatilis* **stat. n.** Galhano, 1968. *Nitocra taylori* **sp. n.** appears to be closely related to *Nitocra husmanni*. Unfortunately, the original description of the micro-characters of the species lacks the detail needed to make reliable comparisons between species of the genus *Nitocra*. The main differences observed are the number of spinules along the posterior margin of the anal operculum, length ratio of the exopod and endopod of the first swimming leg, shape of the outer spine on the male third endopodal segment of the third swimming leg, number of segments of the male antennule, relative length of the setae on the male baseoendopod of the fifth leg, shape of the male exopod of the fifth leg, relative length of the two setae of the male sixth leg, and shape of the female baseoendopod of the fifth leg. The current distribution of *Nitocra taylori* **sp. n.** is limited to the lake part of the estuary, an area which is most severely affected by the current freshwater deprivation crisis. During closed mouth conditions, these regions (South/North Lake and False Bay) are characterized by low water levels, high salinities and high turbidity levels. This suggests that *Nitocra taylori* **sp. n.** may favor these environmental conditions and the significant correlations found between the abundance of *Nitocra taylori* **sp. n.** and salinity and turbidity confirm this to a degree. *Nitocra taylori* **sp. n.** individuals are also able to withstand a wide range of fluctuations. They were recorded at turbidities ranging from 2 to 102 NTU, temperatures from 20.9 to 34.8 ºC and salinity levels ranging from 9.81 to 53.7 psu. However, in the current state of the system, salinity and temperature levels in the northern regions frequently exceed this value. Continued freshwater deprivation may, therefore, further limit the distribution range of this species.

## Introduction

During the course of routine quarterly monitoring surveys undertaken during the last six years in the St Lucia Estuary, South Africa, several specimens of an unidentified harpacticoid copepod were retrieved from zooplankton samples.The St Lucia Estuary is the largest estuarine lake in Africa and has high priority for conservation as it forms part of South Africa’s first World Heritage Site - iSimangaliso (formerly Greater St Lucia) Wetland Park (Fielding et al. 1991, Cyrus and Vivier 2006) and is a Ramsar Wetland of International Importance (Begg 1978).

**Figure 1.**
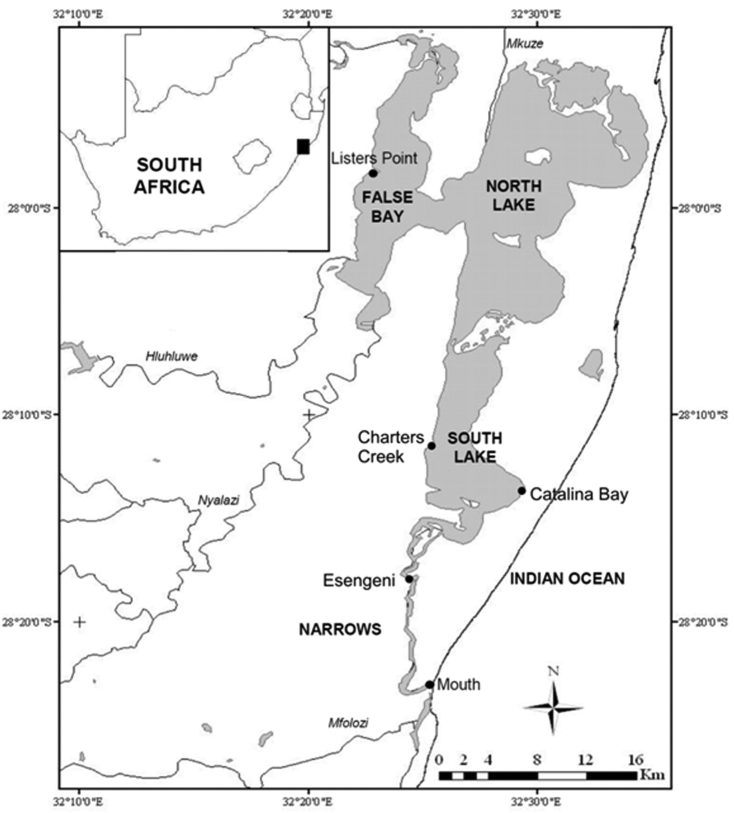
St Lucia Estuary showing the sampling stations and geographic location within South Africa.

St Lucia characteristically experiences cyclical wet and dry phases, each lasting between four and ten years (Begg 1978). The below average rainfall which the area has received since 2002, coupled with a range of anthropogenic interventions undertaken during the last century, have resulted in the current drought cycle being one of the most severe the estuary has ever experienced (Whitfield and Taylor 2009). Low freshwater input and high evaporation rates have led to the persistence of a reversed salinity gradient, with hypersaline conditions in the upper reaches, i.e. False Bay and North/South lakes (Figure 1). The northern lakes are also extremely susceptible to desiccation. At the peak of the drought in 2005–2006, up to 70% of the lake bed was dry (Whitfield and Taylor 2009) and water temperatures in the shallow regions at times reached 50˚C.

In light of the current crisis, research efforts have been directed at clarifying the biodiversity structure of the ecosystem and the effects of this stress on its functioning. A number of new and potentially endemic taxa have been identified recently from samples collected during routine monitoring surveys in the St Lucia Estuary (Daly et al. 2012, Todaro et al. 2011). High densities of an unidentified harpacticoid of the genus *Nitocra*, here described as *Nitocra taylori* sp. n., were collected from South Lake and False Bay. The genus *Nitocra* belongs to the large and heterogenous family Ameiridae. Amerids inhabit a wide range of sediment types and occur in virtually all salinity regimes (Boxshall and Halsey 2004). The first record of *Nitocra taylori* sp. n. in the St Lucia Estuary dates back to 2006, when low densities were collected from Charters Creek, which is situated on the western shore of South Lake. It is possible that this species was present in earlier assessments conducted by Grindley (1976), however, in these assessments, harpacticoid copepods were not identified to species level. This study, therefore, aims to describe this species as well as document the basic characteristics of the habitat in which it occurs, in order to provide management with knowledge for the adequate protection of this potentially endemic species.

## Material and methods

Quarterly surveys were undertaken at five representative stations within the St Lucia Estuary, from February 2006 through to May 2011. These stations included the Mouth, Esengeni, Catalina Bay, Charters Creek and Listers Point (Figure 1). The study period covered three different hydrological phases, viz. a closed-mouth phase (February 2006 to February 2007), an open-mouth phase (March 2007 to August 2007), and a re-closed period (November 2007 to May 2011). Zooplankton together with physico-chemical data, were collected at each site on each sampling occasion.

### Physico-chemical variables

Physico-chemical measurements were taken with a YSI 6920 water quality logger, fitted with temperature (˚C), depth (m), conductivity (mS/cm), dissolved oxygen (mg.L^-1^), pH and turbidity (Nephelometric Turbidity Units or NTUs) probes.

### Zooplankton sampling

Single daytime mesozooplankton samples were collected using an epibenthic sled (100 μm mesh). The sled was towed in the shallow waters near-shore at all stations, except at Esengeni, where a boat was used. The mouth of the net was semi-circular in shape (radius = 18.5 cm) and was mounted on a sled that was towed just above the sediment surface. Sampling with this sled, therefore, allowed the suitable collection of epibenthic harpacticoids. In regions where water depth was too shallow, or when zooplankton was too dense for the sled to be used, 30 L of water was passed through a 100 *µ*m sieve. Samples were emptied into 500 mL bottles containing 4% phloxine-stained formaldehyde.

In the laboratory, samples were suspended in 0.5 to 5 L solutions, depending on the density of organisms. The main sample was then stirred vigorously so that all the organisms remained in a homogenous suspension. A 20 mL plastic vial attached to a metal rod was then used to withdraw three subsamples from mid-depth (Perissinotto and Wooldridge 1989, Carrasco et al. 2010). Individuals of *Nitocra taylori* sp. n. within the samples were identified and counted with a Kyowa 129 SDZ dissecting microscope (400 x) and density was then calculated as ind.m^-3^.

### Statistical analyses

Univariate statistical analyses were conducted with SPSS version 19 for Windows, where a Pearson correlation was used to test for relationships between environmental variables and species abundance. This analysis was performed on log-transformed abundance data for all non-zero records.

### Species description

A number of adult males and females were fixed in 4% formalin and preserved in 70% ethanol for taxonomic analyses and description. Observations and drawings were done at a magnification of 1000 x from whole and dissected specimens mounted in lactophenol with a Leica compound microscope equipped with phase contrast and a drawing tube. The type material was deposited in the collection of the Instituto de Ciencias del Mar y Limnología, Unidad Académica Mazatlán (Mexico) (EMUCOP) and in the collection of the Iziko South African Museum, Cape Town (South Africa) (SAM). The terminology proposed by Huys and Boxshall (1991) for the general description was adopted. The relative length of the setae of the male and female P5 was calculated dividing the length of each seta by the total length of the longest element. Abbreviations used in the text and tables are: P1-P6, first to sixth swimming legs; EXP, exopod; ENP, endopod; P1(P2-P4)EXP(ENP)1(2, 3) denotes the proximal (middle, distal) exopodal (endopodal) segment of P1, P2, P3 or P4; BENP, baseoendopod; ae, aesthetasc.

## Taxonomic account

### Family Ameiridae Monard, 1927

Subfamily Ameirinae Lang, 1944

Genus *Nitocra* Boeck, 1865

#### Nitocra taylori sp. n.

urn:lsid:zoobank.org:act:AF4F4062-2DF8-41B5-9089-B62EECAB56B1

http://species-id.net/wiki/Nitocra_taylori

**Figure 2.**
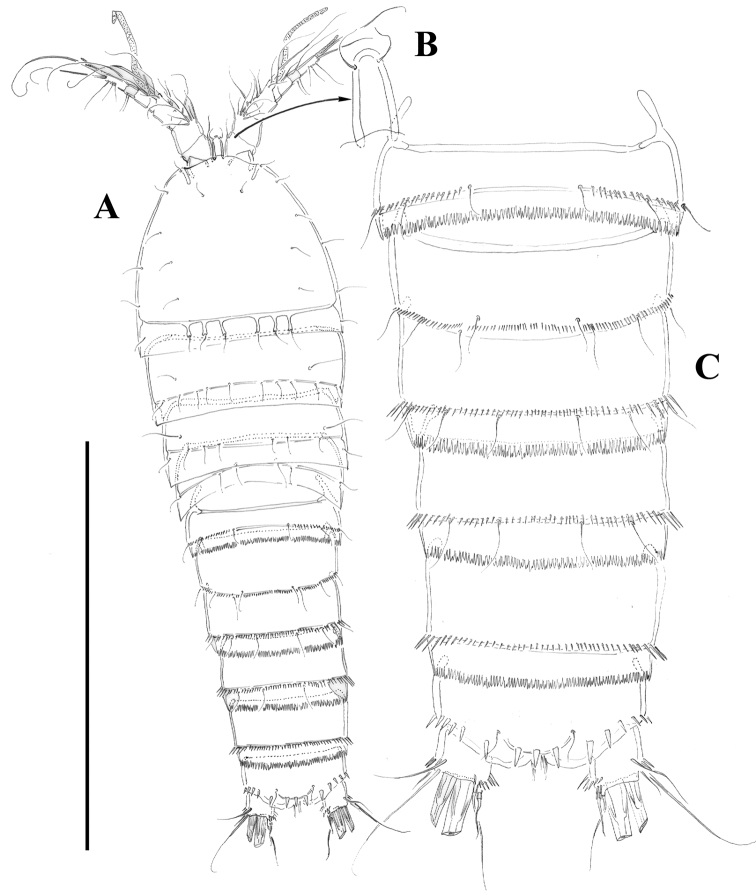
*Nitocra taylori* sp. n. Female. **A** habitus **B** rostrum, dorsal **C** urosome, dorsal. Scale bar: **A**=300 µm; **B**=75 µm; **C**=150 µm.

Figures 2 [Fig F3] [Fig F4] [Fig F5] [Fig F6] [Fig F7] [Fig F8] 9

##### Type material.

One female holotype (EMUCOP-080311-03) and one male allotype (EMUCOP-080311-06) preserved in alcohol, one female (EMUCOP-080311-04) and one male (EMUCOP-080311-05) dissected paratypes, and 15 adult females, 11 adult males, one CIII, three CIV and three CV paratypes preserved in alcohol (EMUCOP-080311-07) were deposited in the Copepoda collection of the Instituto de Ciencias del Mar y Limnología, Mazatlan Marine Biological Station; 26 additional paratypes (SAM-A45750) were deposited in the collection of the Iziko South African Museum; collected from Listers Point, St Lucia Estuary, South Africa; 8 March 2011; leg. N. K. Carrasco.

##### Type locality.

Listers Point, False Bay, St Lucia Estuary, South Africa (27º58'09.4"S, 32º22'48.11"E).

##### Etymology.

The species is named after Dr Ricky H. Taylor, former Regional Ecologist at Ezemvelo KZN Wildlife, St Lucia Estuary, for his invaluable help provided to research and his lifetime efforts towards the conservation of the St Lucia Estuary. The specific epithet is a noun in the genitive singular.

**Figure 3. F3:**
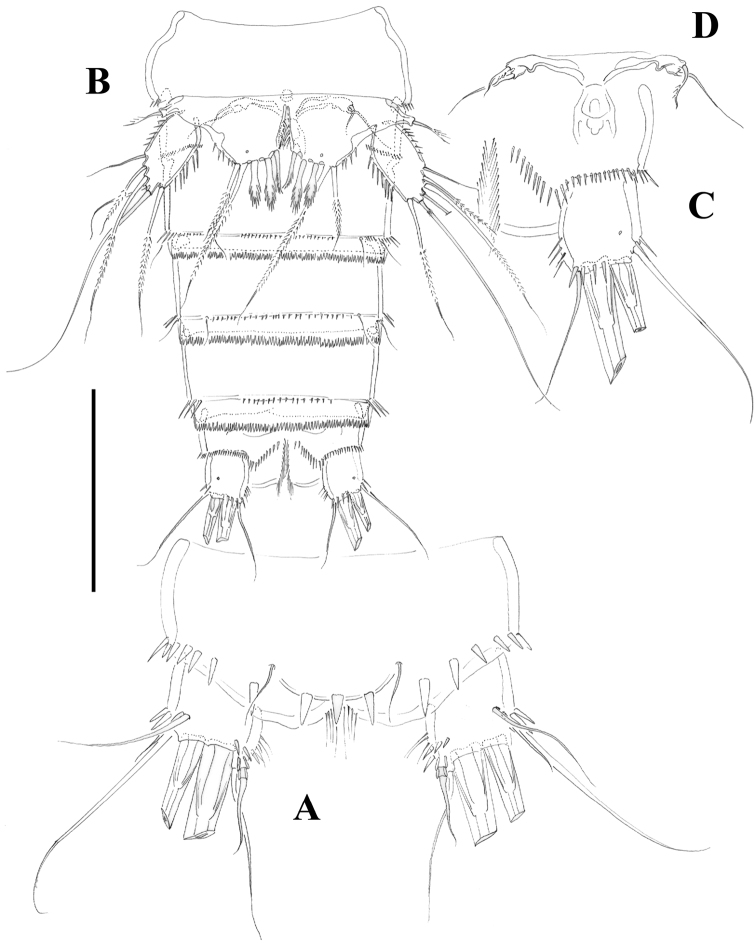
*Nitocra taylori* sp. n. Female. **A** anal somite and caudal rami, dorsal **B** urosome, ventral, showing P5 **C** left caudal ramus, ventral **D** P6 and genital complex. Scale bar: **A**=44 µm; **B**=100 µm; **C**=50 µm; **D**=71 µm.

Female.Habitus (Figure 2A) tapering posteriorly; total body length measured from tip of rostrum to posterior margin of caudal rami ranging from 460 to 685 µm (mean, 537 µm; n= 11; holotype, 525 µm). Rostrum (Figure 2A, B) defined at base, elongate, small, barely reaching distal margin of first antennulary segment, with pair of sensilla subapically. Dorsal surface of cephalic shield and free prosomites without spinular ornamentation, with plain caudal frill (Figure 2A). P5-bearing somite with medially interrupted row of minute spinules close to posterior margin dorsolaterally, with deeply serrate caudal frill (Figure 2A, C). Subcuticular rib of genital double-somite with dorsolateral row of small spinules indicating former division between second and third urosomites (Figure 2A, C), but completely fused ventrally (Figs 3B, 10F); third urosomite with comparatively stronger spinules close to posterior margin dorsally and laterally, with median row of minute spinules ventrally, with deeply serrate caudal frill. Fourth and fifth urosomites as previous somite dorsally and ventrally, except for less and lack of sensilla in fourth and fifth urosomites, respectively. Anal somite somewhat shorter than previous somite, with strong spinules dorsally and laterally close to joint with caudal rami (Figs 2A, C, 3A), with comparatively smaller spinules ventrally (Figs 3B, C, 10F); rounded anal operculum with three strong spinules close to posterior margin, and flanked by pair of sensilla (Figs 2C, 3A). Caudal rami nearly as long as wide from dorsal view, but slightly longer than wide ventrally (Figs 2C, 3A, B, C, 10B), with seven setae as follows: seta I small, nearly as long as caudal ramus; seta II dorsal to seta I, about twice as long as the latter; seta III about twice as long as seta II, arising close to outer distal corner; setae IV and V well developed, the latter longest; seta VI arising from inner distal corner, slightly shorter than seta III; seta VII biarticulated, rather short, arising close to base of seta VI at inner distal corner.

Antennule (Figure 5A) eight-segmented, surface of segments smooth except for spinular row on first segment. Armature formula as follows: 1-(1), 2-(9), 3-(8), 4-(3 + [1+ae]), 5-(2), 6-(3); 7-(4); 8-(5+acrothek). Fourth segment with one outer spinule. Acrothek consisting of two setae and one aesthetasc fused at their base.

**Figure 4. F4:**
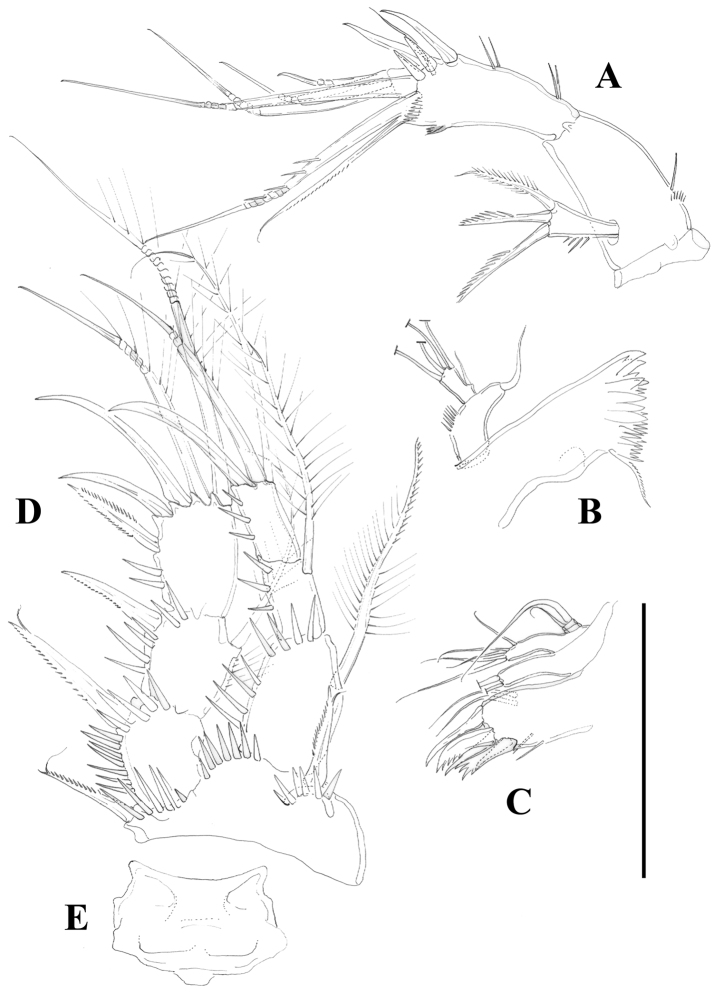
*Nitocra taylori* sp. n. Female. **A** antenna **B** mandible **C** maxillule **D** P1, anterior **E** intercoxal sclerite of P1, anterior. Scale bar: **A–E**=50 µm.

Antenna (Figure 4A) with small coxa. Allobasis without abexopodal setae, and ornamented with one long spinule and a short row of minute spinules proximally. Free endopodal segment with inner spinules proximally and subdistally, with two lateral inner spines and one slender seta, and four single geniculate setae and one geniculate element fused basally to pinnate seta. Exopod one-segmented; with few spinules, and three setae (two pinnate spine like elements and one bipinnate seta).

Mandible (Figure 4B) robust; gnathobase with bi- and multicuspidate teeth, and one lateral seta. Mandibular palp two-segmented; first segment (basis) with some spinules and one seta; second segment (endopod) with one lateral and four apical setae.

Maxillule (Figure 4C). Arthrite of praecoxa with few spinules, with two surface setae, and three bare spines and two serrate/multicuspidate elements. Coxa with two elements. Basis seemingly with four setae; exopod vestigial represented by one seta; endopod two-segmented, first segment without any setae, second segment with two setae.

Maxilla (Figure 6A). Syncoxa with minute outer spinules; with one endite bearing three setae. Allobasis drawn into strong claw with one accompanying strong element. Endopod one-segmented, with two setae.

Maxilliped (Figure 6B) subchelate. Syncoxa with spinular rows and with one seta on inner distal corner. Basis unarmed, with longitudinal row of spinules, with some outer spinules distally. Endopod drawn into long and slender claw with one accompanying small seta.

P1 (Figure 4D, E). Intercoxal sclerite without spinular ornamentation; distal margin convex. Basis with inner and outer flagellate spine; with strong spinules at base of inner spine, between rami and at base of exopod. Exopod and endopod three-segmented; EXP1 without inner seta, EXP2 with plumose inner seta; EXP3 with one outer proximal bipinnate spine, two outer naked spines and two geniculate elements. Endopod three-segmented; slightly beyond EXP; first segment slightly shorter that second and third segments combined, reaching insertion level of inner seta of EXP2; first segment with inner seta ornamented medially with setules and with spinules along outer margin distally; second segment with plumose inner seta; third segment with one inner plumose seta apically, one median geniculate element, and one apical outer spine. Armature formula as below.

**Figure 5. F5:**
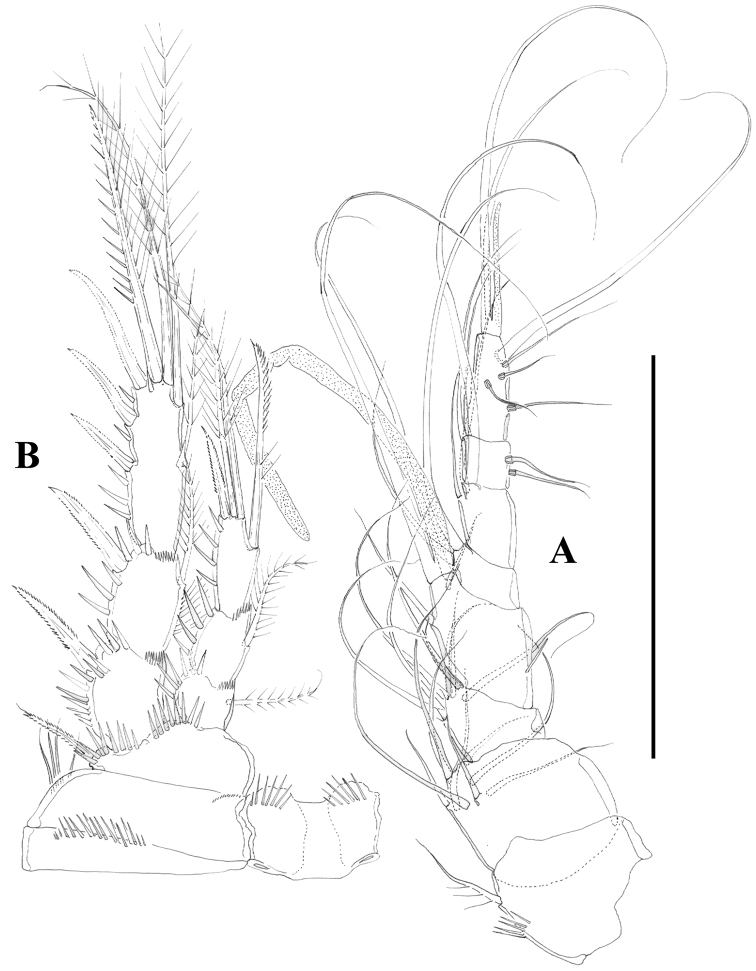
*Nitocra taylori* sp. n. Female. **A** antennule **B** P2, anterior. Scale bar: **A**=70 µm; **B**=100 µm.

P2 (Figure 5B). Intercoxal sclerite with transverse spinular row distally on both lobes. Praecoxa with spinules close to joint with coxa. The latter with long outer setules and minute spinules close to outer and inner distal corner, respectively. Basis with outer spine; with strong spinules between rami and at base of EXP. Exopod three-segmented; first segment without setae, second segment with plumose inner seta; third segment with three outer bipinnate spines, one outer apical seta ornamented with strong spinules and setules along outer and inner margin, respectively, one inner apical plumose seta and one inner plumose seta. Endopod three-segmented, reaching proximal fourth of EXP3; first and second segments with inner plumose and short seta; third segment with one strong inner seta ornamented with few setules medially and with spinules along outer margin distally, two apical plumose setae and one outer bipinnate spine. Armature formula as below.

**Figure 6. F6:**
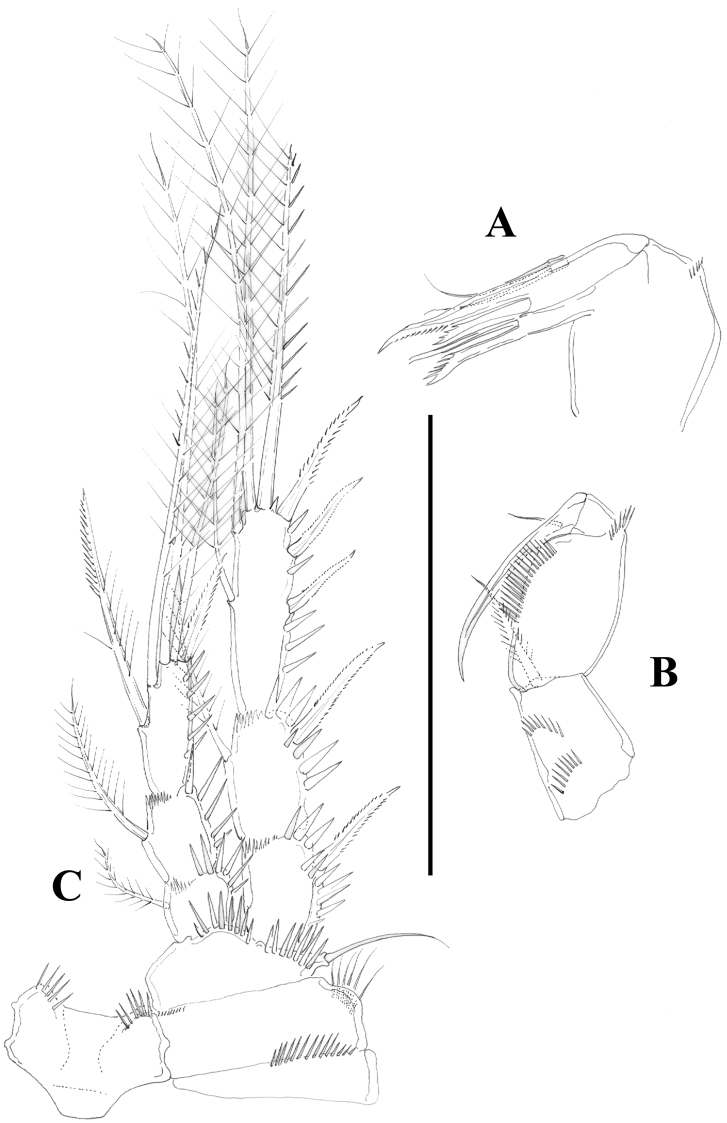
*Nitocra taylori* sp. n. Female. **A** maxilla **B** maxilliped **C** P3, anterior. Scale bar: **A, B**=70 µm; **C**=100 µm.

P3 (Figure 6C). Intercoxal sclerite, praecoxa and coxa as in P2. Basis as in P2 except for outer seta-like element in P3. Exopod as in P2. Endopod as in P2 except for additional inner element in P3ENP3 ornamented with setules proximally and with spinules along outer margin distally; reaching proximal third of EXP3.

**Figure 7. F7:**
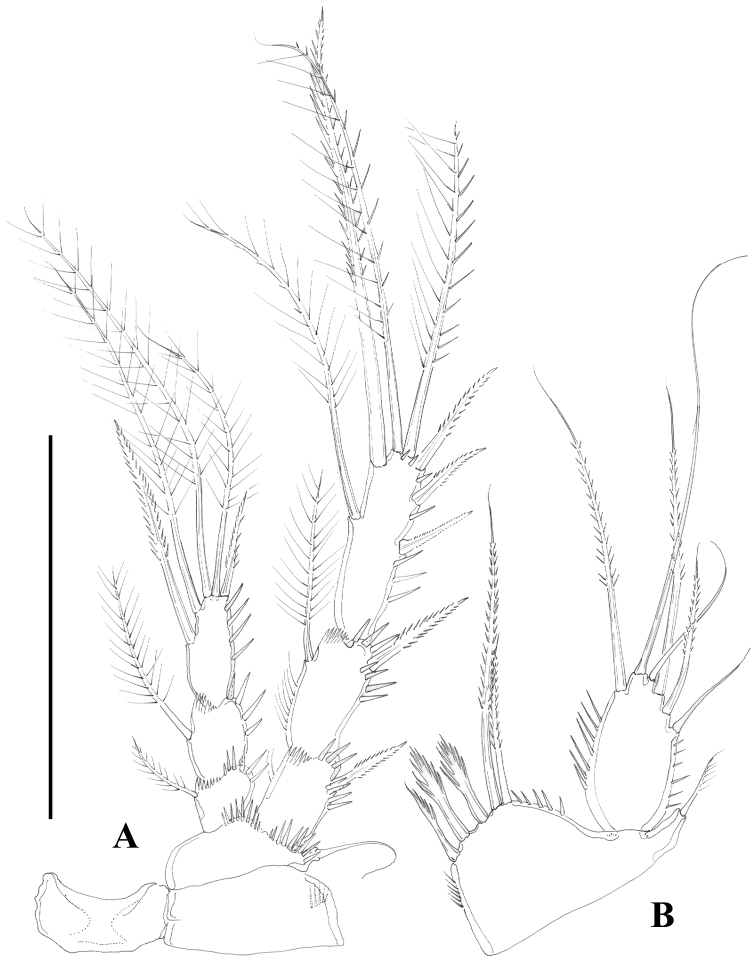
*Nitocra taylori* sp. n. Female. **A** P4, anterior **B** P5, anterior. Scale bar: **A, B**=100 µm.

P4 (Figure 7A). Intercoxal sclerite without spinules. Praecoxa (not shown), coxa and basis as in P3. Exopod as in P3 except for comparatively stronger inner distal seta of P4EXP3 ornamented with outer and inner spinules, and for outer apical seta ornamented with inner setules and outer spinules. Endopod as in P3, except for bipinnate inner proximal seta on P4ENP3; slightly beyond EXP2.

P5 (Figure 7B). Both legs separated. Exopod and baseoendopod not fused. Exopod ovate; with inner and outer spinules; with six elements; relative length of the setae from inner to outer element as follows: 0.73, 1, 0.38, 0.69, 0.38, 0.25. Endopodal lobe with five setae/spines; relative length of the setae from inner to outer element as follows: 0.30, 0.31; 0.30, 1, 0.52; with inner and outer spinules.

Armature formula of female P1-P5 as follows:

P6 (Figure 3D) represented by median plate in anterior half of second urosomite (first genital somite); each vestigial leg represented by one outer short and one inner long seta.

**Figure 8. F8:**
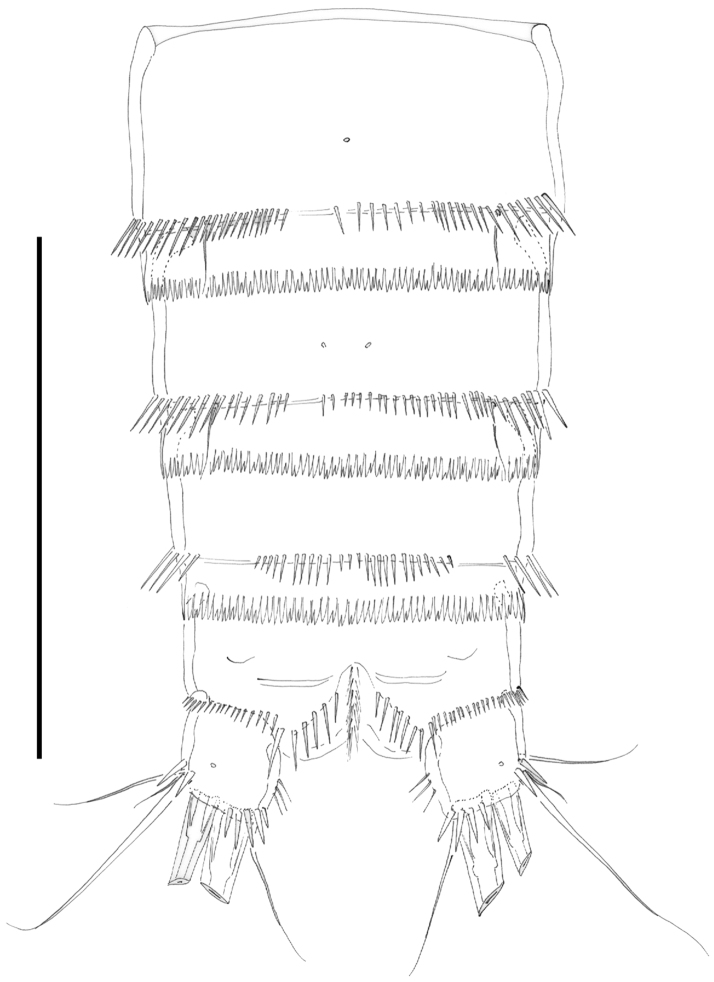
*Nitocra taylori* sp. n. Male. Urosome, ventral (P5- and P6-bearing somites omitted). Scale bar: 100 µm.

Male. Habitus (not shown) as in female, except for distinct second and third urosomites; total body length measured from tip of rostrum to posterior margin of caudal rami ranging from 385 to 520 µm (mean, 437 µm; n= 7; allotype, 490 µm). Ventral spinular ornamentation of third-sixth urosomites coarser and stronger than in female (Figs 8, 10D). Caudal rami (Figs 8, 10D) as in female.

Sexual dimorphism expressed in the antennule, P1Basis, P3ENP, P5 and P6.

**Figure 9.**
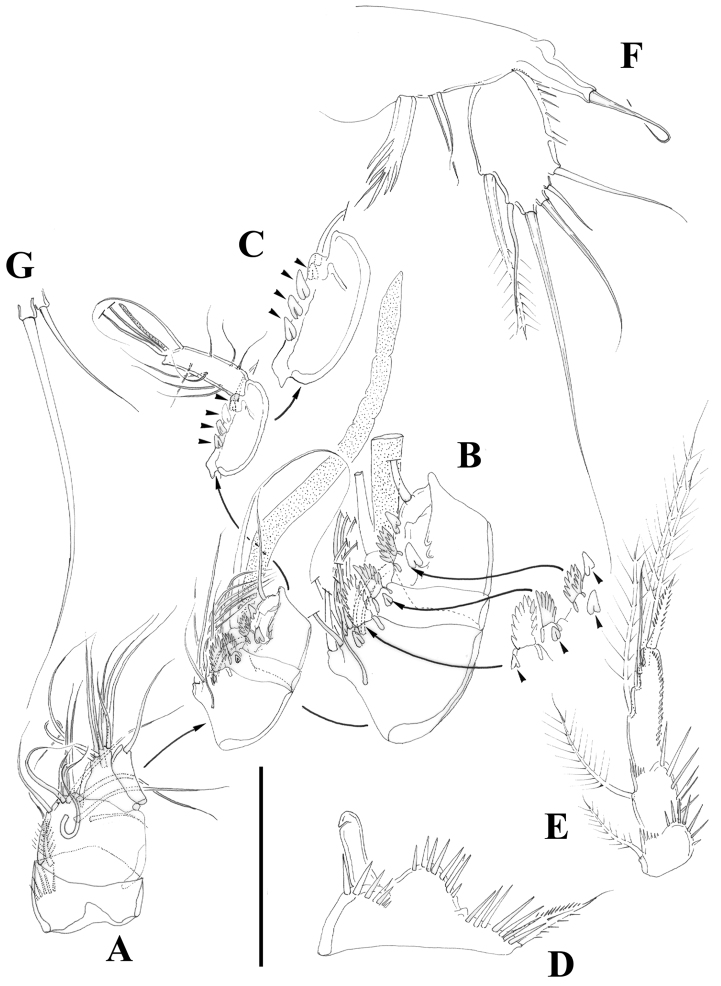
*Nitocra taylori* sp. n. Male. **A** antennule **B** fifth, sixth and seventh segments of the antennule, showing modified setae and blunt processes **C** eight segment of the antennule **D** P1 basis, anterior **E** P3ENP **F** P5, anterior **G** P6, anterior. Scale bar: **A, E**=50 µm; **B, C**=67 µm; **D, F, G**=35 µm.

Antennule (Figure 9A) haplocer, nine-segmented; armature formula difficult to define, but most probably as follows: 1-(1), 2-(11), 3-(8), 4-(1), 5-(14+[1+ae]), 6-(1), 7-(2); 8-(1); 9-(9+acrothek); fifth, sixth and seventh (Figure 9B) and eight (Figure 9C) segments with modified setae and blunt spines/processes. Acrothek consisting of two setae and one aesthetasc basally fused.

**Figure 10.**
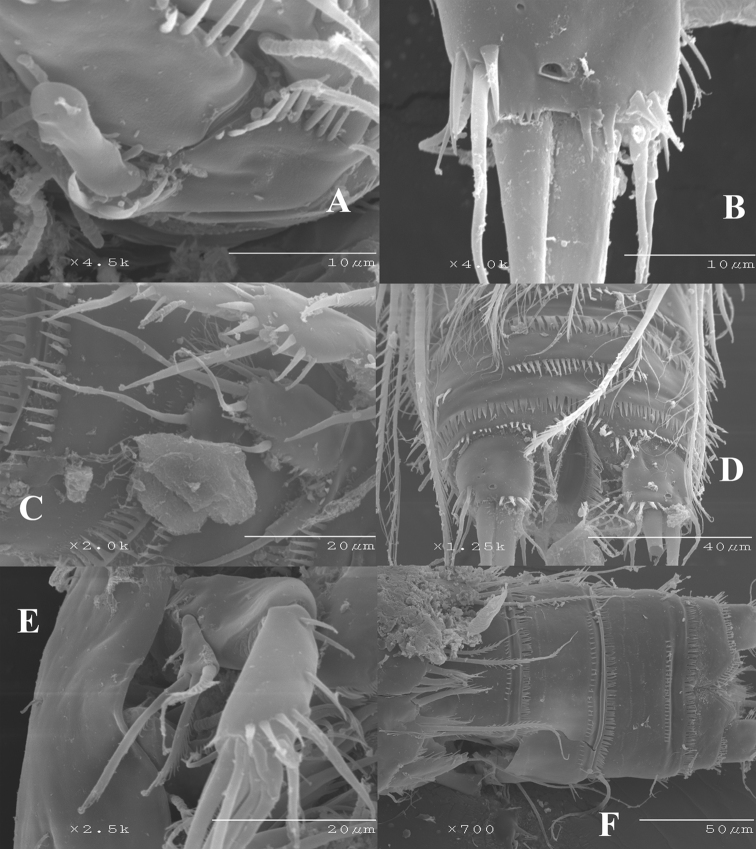
*Nitocra taylori* sp. n. SEM photographs **A** male P1 showing inner modified spine of basis **B** female caudal rami **C** male P5 exopod and P6 **D** posterior part of male urosome including caudal rami **E** male antenna **F** anterior part of female urosome.

Antenna (Figure 10E), mandible, maxillule, maxilla and maxilliped (not shown) as in female.

P1-P4 as in female, except for sexually dimorphic male P1 Basis (Figs 9D, 10A) and P3ENP (Figure 9E). The former with modified inner spine. The latter three-segmented; first and second segment as in female; third segment with outer longitudinal row of small spinules and armed with five setae/spines.

P5 (Figs 9F, 10C). Both legs fused medially. Exopod and baseoendopod separated. The former ovate, with six setae, relative length of elements from inner to outer margin as follows: 0.47, 0.35, 1.0, 0.44, 0.17, 0.39. Baseoendopodal lobe poorly developed, with three elements; relative length of elements from inner to outer margin as follows: 1.0, 0.79, 0.67.

P6 (Figs 9G, 10C) represented by two setae situated rather laterally, outer seta smaller than inner element.

##### Ecology.

**Habitat characteristics.** During closed-mouth conditions, the St Lucia Estuary was characterised by a reversed salinity gradient, with salinities ranging from near freshwater conditions at the Mouth and Narrows, to up to 200 psu at times in the northern regions of the lake (Table 1). Salinity levels were also more variable in the lakes than in the Mouth and Narrows region. Throughout the study period, salinity levels at the Mouth ranged from 3.2 to 37.6 psu, while at Listers Point they ranged from 18.3 to 216 psu. During closed-mouth conditions, water depth was also generally highest at the Mouth and Esengeni and shallower (~0.2 m) in the lakes (Table 1). High wind action, coupled with the fine sandy substratum in the lakes, also resulted in higher turbidity levels experienced here relative to the Mouth and Narrows. Listers Point and Charters Creek generally experienced the highest turbidities, while levels at the Mouth were usually at least one order of magnitude lower (Table 1). During open-mouth conditions, there was little disparity between sites in terms of the physico-chemical parameters measured. Salinity was generally within the range of sea water (~35 psu) across the estuarine lake and water levels in the lakes rose to approach the levels recorded at the Mouth and Narrows (Table 1).

##### *Nitocra taylori* sp. n. abundance and distribution.

**Figure 11.**
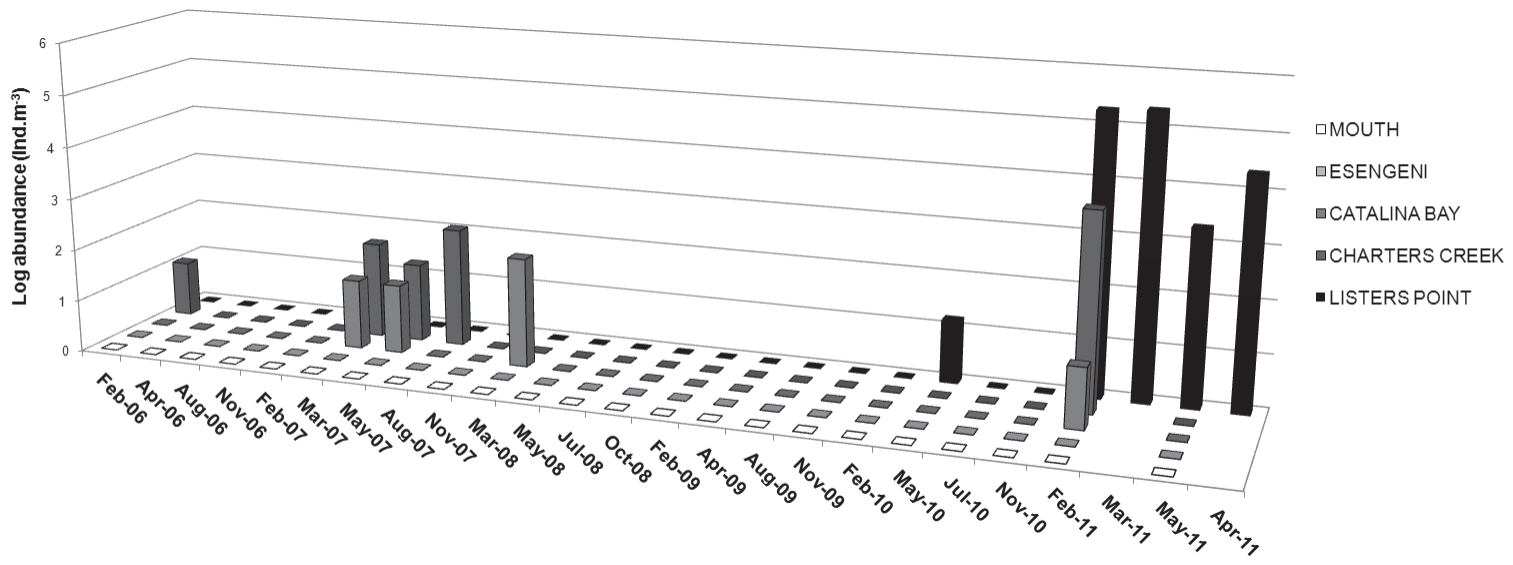
Abundance of *Nitocra taylori* sp. n. (ind.m^-3^) in the St Lucia Estuary from February 2006– April 2011.

**Figure 12.**
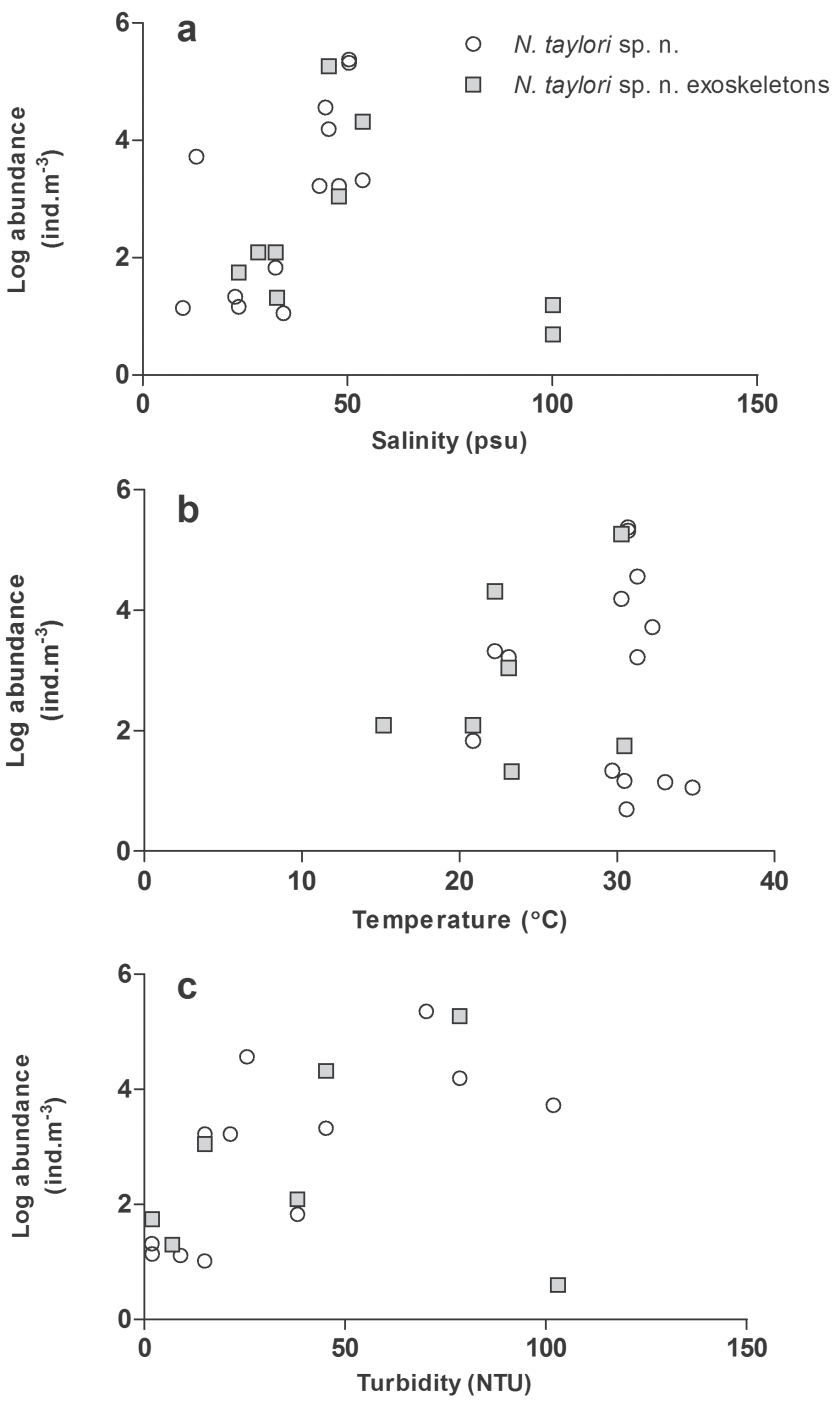
Abundance of *Nitocra taylori* sp. n. (alive and discarded exoskeletons) at different **a** salinity **b** temperature and **c** turbidity levels recorded in the St Lucia Estuary.

Occurrence of *Nitocra taylori* sp. n. through the study years has been irregular and the distribution has been limited to Catalina Bay and Charters Creek in South Lake and Listers Point in False Bay (Figs 1, 11). *Nitocra taylori* sp. n. was first recorded at Charters Creek in February 2006 in low densities (10.4 ind.m^-3^), while maximum densities (2.2 x 10^5^ ind.m^-3^) were recorded at Listers Point in March 2011. These high densities followed heavy dilution of hypersaline waters after high rainfall in early 2011. Densities remained high in this region up until May 2011, after which salinities rose again above 53.7 psu and *Nitocra taylori* sp. n. virtually disappeared. Correlation analysis found significant positive correlations between the abundance of *Nitocra taylori* sp. n. and salinity (R = 0.621, p < 0.05, df = 12) and turbidity (R = 0.681, p < 0.05, df = 12). *Nitocra taylori* sp. n. individuals were able to withstand a wide range of fluctuations. They were found at salinity levels ranging from 9.81 to 53.7 psu, turbidities ranging from 2 to 102 NTU and temperatures from 20.9 to 34.8 ºC (Figure 12). In many instances specimens preserved in phloxine-stained formaldehyde did not take up the stain, but were rather completely transparent, resembling discarded exoskeletons. While these individuals were perfectly intact, it is unlikely that they were alive at the time of collection.

**Table 1.** Physicochemical variables measured at each station during the different mouth phases (mean ± SE). NTU: Nephelometric Turbidity Units; DO: dissolved oxygen.

**Mouth state**	**Station**	**Depth(m)**	**DO(mg.^L-^1)**	**pH**	**Salinity(psu)**	**Temperature(ºC)**	**Turbidity(NTU)**
Closed	Mouth	2.05 ± 0.52	7.9 ± 0.52	8.02 ± 0.37	12.8 ± 1.67	26.5 ± 2.32	9.36 ± 5.82
	Esengeni	1.38 ± 0.12	7.75 ± 1.13	8.37 ± 0.17	6.45 ± 1.52	24.5 ± 1.98	28.7 ± 3.76
	Catalina Bay	0.15 ± 0.04	9.06 ± 0.91	8.52 ± 0.14	14.5 ± 3.24	24.8 ± 2.58	42.8 ± 20.5
	Charters Creek	0.11 ± 0.02	8.38 ± 1.04	8.79 ± 0.22	24 ± 4.48	29.2 ± 2.5	33.2 ± 11.0
	Listers Point	0.07 ± 0.02	4.96 ± 1.52	8.17 ± 0.14	43.9 ± 21.58	29.8 ± 1.44	49.6 ± 12.7
Open	Mouth	0.75 ± 0.35	7.57 ± 0.63	8.08 ± 0.14	34.6 ± 1.05	23.1 ± 1.6	24.4 ± 15.8
	Esengeni	1.51 ± 0.21	5.23 ± 1.48	8.25 ± 0.07	37.4 ± 9.38	20.3 ± 2.98	45.6 ± 13.5
	Catalina Bay	0.33 ± 0.18	7.5 ± 1.26	7.99 ± 0.38	29.1 ± 3.26	23.7 ± 3.4	8.37 ± 4.18
	Charters Creek	0.53 ± 0.22	7.82 ± 1.52	8.11 ± 0.21	29.6 ± 3.1	24.5 ± 3.63	13.4 ± 5.96
	Listers Point	0.43 ± 0.28	6.91 ± 0.91	7.01 ± 1.01	35.1 ± 4.25	23.8 ± 2.73	32.8 ± 17.7
Re-closed	Mouth	1.4 ± 0.23	6.85 ± 1.02	8.72 ± 0.26	15.3 ± 2.44	23.0 ± 0.87	20.0 ± 3.68
	Esengeni	1.35 ± 0.13	7.51 ± 0.58	8.36 ± 0.17	9.39 ± 2.19	23.3 ± 0.81	60.3 ± 12.6
	Catalina Bay	0.21 ± 0.04	7.01 ± 1.18	8.61 ± 0.26	37.5 ± 5.25	24.8 ± 1.23	50.6 ± 10.5
	Charters Creek	0.2 ± 0.04	7.51 ± 0.39	8.65 ± 0.19	45.3 ± 5.3	26.3 ± 1.06	197 ± 61.2
	Listers Point	0.31 ± 0.05	7.53 ± 0.58	8.53 ± 0.19	87.4 ± 12.8	25.6 ± 1.73	155 ± 41.1

## Discussion

### Taxonomic remarks

The phylogenetic relationships of the species within the genus *Nitocra* are still obscure. At first glance, three species groups can be recognized based on the combination of the armature formula of the P1EXP2-3. The less speciose group, composed only by *Nitocra sewelli* Gurney, 1927 and *Nitocra platypus bakeri* Chappuis, 1930, exhibits one inner seta and four elements on the P1EXP2 and EXP3 respectively. This two-species group is followed by that lacking inner armature on the P1EXP2, but with five setae on P1EXP3 (*Nitocra reducta* s. str. (Schäfer 1936), *Nitocra delaruei* Soyer, 1974, and *Nitocra blochi* Soyer, 1974). The rest of the species/subspecies belongs to the most diverse and seemingly primitive group characterized by the presence of one inner, and five elements on the P1EXP2 and EXP3, respectively.

The creation of species and subspecies of Harpacticoida might appear, sometimes, based on questionable grounds. This holds true, as evidenced by Wells and Rao (1987), for *Nitocra spinipes* s. str. Boeck, 1865, *Nitocra spinipes orientalis* Sewell, 1924 and *Nitocra spinipes armata* Lang, 1965. The same applies at least for the subspecies of *Nitocra reducta* and *Nitocra sewelli*. As evidenced upon a brief analysis, the subspecific relationship of *Nitocra reducta* s. str. and *Nitocra reducta fluviatilis*Galhano, 1968 is questioned given the lack of inner armature in the former and the presence of an inner element in the latter. In our opinion, such difference is not evidence of the presence of a subspecies of *Nitocra reducta*, but of a different species. Galhano (1968) herself noted also a remarkable difference in the armature formula of the female P5 baseoendopod (with four setae in Schäfer’s *Nitocra reducta*, but with five elements in Galhano’s material). In the view of this evidence it is suggested to grant *Nitocra reducta fluviatilis*Galhano, 1968 full species rank as *Nitocra fluviatilis* stat. n. Galhano, 1968, until the variability of these two species is properly assessed.

A similar case was observed for *Nitocra sewelli*. Sewell (1924) described a new variety of the European *Nitocra typica* Boeck, 1865, *Nitocra typica lacustris* Sewell, 1924. However, the name *lacustris* was already occupied by *Nitocra lacustris* (Schmankevitch 1875) and Gurney (1927) renamed and gave full species rank to *Nitocra typica lacustris* as *Nitocra sewelli*. He also presented a key to species of the genus in which he recognized 11 members as valid (*Nitocra pusilla* Sars, 1911, *Nitocra inuber* (Schmankevitch, 1875), *Nitocra affinis*, *Nitocra typica*, *Nitocra dubia* Sars, 1927, *Nitocra lacustris*, *Nitocra fragilis* Sars, 1905, *Nitocra wolterecki* Brehm, 1909, *Nitocra spinipes*, *Nitocra sewelli* and *Nitocra platypus* Daday, 1906. Lang (1948) recognized 16 valid species and 7 subspecies, and relegated *Nitocra inuber* (=*Dactylopus inuber* Schmankevitsch, 1875), *Nitocra gracilimana* Giesbrecht, 1902, *Nitocra wolterecki*, *Nitocra phlegraea* Brehm 1909, and *Nitocra chelifer* Wilson, 1932, as *incertae sedis* within the genus. Kunz (1976) described a new subspecies of *Nitocra sewelli*, *Nitocra sewelli husmanni* Kunz, 1976 from Bremen (Germany). In his analysis, Kunz (1976) noted that *Nitocra sewelli husmanni* showed the same armature formula as in *Nitocra spinipes* s. str., *Nitocra spinipes orientalis*, *Nitocra spinipes armata* (not *armatus* as in Kunz (1976); note that these subspecies were rejected by Wells and Rao 1987, and were not considered in Wells (2007)), *Nitocra elegans* (T. Scott, 1905), *Nitocra fragilis*, *Nitocra bdellurae* (Liddell, 1912), *Nitocra sewelli* and *Nitocra medusae* Humes, 1953 (which was considered synonym of *Nitocra spinipes* by Lang (1965)). However, as shown by Sewell (1924), the P1EXP3 of *Nitocra sewelli* is armed with four setae/spines instead of five elements as in the rest of the above species. Kunz (1976) observed some other differences between his material and Sewell´s (1924) description of *Nitocra sewelli*, such as the number of setae on the male P5BENP, relative length of the setae of the male and female P5EXP and shape of the outer dimorphic spine on the male P3ENP3. Sewell (1924) found two males and one female and nothing is said about the intraspecific variability of the species, which for other species seems to be important in subspecies acceptance or rejection (i.e. Wells and Rao 1987). On the other hand, Kunz (1976) found some variability mainly expressed in the armature formula of the female P2ENP2 and P3EXP3, and in the relative length of the setae on the male P5BENP, but the armature formula of the P1EXP remains constant. It seems, therefore, unlikely that the armature formula of P1EXP3 observed by Sewell (1924) in the Indian material is due to intraspecific variability, which, by the way, was not observed by Kunz (1976) in the German specimens. In our opinion, Kunz (1976) erected his new subspecies based more on the differences with some other species, than on the similarities with *Nitocra sewelli* (to which it is implicitly assumed to be more closely related), being the different armature formula of P1EXP and the differences observed by Kunz (1976: 33) enough to separate *Nitocra sewelli* from *Nitocra sewelli husmanni*. It is, therefore, suggested that the latter be granted full species rank as *Nitocra husmanni stat. n*. Kunz 1976.

At present and taking into account the rejection of all the described subspecies of *Nitocra spinipes* by Wells and Rao (1987), as well as the amendments above, 45 species within the genus are considered as valid. Of these, eight species (*Nitocra affinis*, *Nitocra divaricata* Chappuis, 1923, *Nitocra fallaciosa* Klie, 1937, *Nitocra hibernica* (Brady, 1880), *Nitocra lacustris*, *Nitocra mediterranea* Brian, 1928, *Nitocra minor* Willey, 1930, *Nitocra platypus*) contain 22 subspecies (Wells 2007). Also, the same species as in Lang (1948) (except for *Nitocra phlegraea* which does not appear in Wells’ (2007) list of species) plus *Nitocra hyperidis* Jakobi, 1956, are regarded as *incertae sedis* within the genus (Lang 1965, Wells 2007).

Within the most speciose group (see above), *Nitocra australis* Soyer, 1974, *Nitocra fragilis*, *Nitocra spinipes*, *Nitocra intermedia* Pesce, 1983, and *Nitocra husmanni* are unique in the combination of the number of setae/spines of the P2-P4Enp3 and P2-P4EXP3 (4,5,5 and 7,7,7, respectively) and number of inner setae on the P2-P4Enp1 (1,1,1). Note that Scott (1905) and Monard (1935) described the P4EXP3 with 7 setae/spines. In his description of the male and amendments to the description of the female of *Nitocra elegans*, Gee (2009) showed that the P4EXP3 possesses in fact eight setae/spines (three inner setae, two apical elements and three outer spines), being the distalmost inner seta very slender and relatively short, implicitly suggesting that this seta might have been overlooked in previous descriptions, being *Nitocra elegans* probably more related to the *Nitocra affinis* complex of subspecies and to *Nitocra hamata* Bodin, 1970.

The South African material herein described agrees well with the description of *Nitocra husmanni* by Kunz (1976), to which it seems to be closely related. Unfortunately, Kunz (1976) description lacks the detail needed to make reliable comparisons between species of the genus *Nitocra*. On the other hand, due to some restrictions regarding the import/export permits of biological material, we were unable to check the specimens of *Nitocra husmanni* deposited by Kunz (1976) in the collection of the Zoologischen Museum Hamburg under catalogue numbers K-30399 and K-30400. The main differences observed between the South African material and *Nitocra husmanni* as described by Kunz (1976), are the number of spinules along the posterior margin of the anal operculum, which is variable in *Nitocra spinipes* (Wells & Rao, 1987) (about seven spines in *Nitocra husmanni*, but three in *Nitocra taylori* sp. n.) ; P1EXP:ENP length ratio (exopod shorter than endopod in *Nitocra husmanni*, but exopod nearly as long as endopod in *Nitocra taylori* sp. n.), shape of the outer spine on the male P3ENP3 (curved in *Nitocra husmanni*, but straight in *Nitocra taylori* sp. n.), number of segments of the male antennule (10 segments in *Nitocra husmanni*, but only nine in *Nitocra taylori* sp. n.), relative length of the setae on the male P5BENP (innermost element visibly shorter than the two adjacent outer setae in *Nitocra husmanni*, but innermost element longer in *Nitocra taylori* sp. n.), shape of the male P5EXP (comparatively more elongate in *Nitocra taylori* sp. n.), relative length of the two setae of the male P6, shape of the female P5BENP (broader, less developed and barely reaching the proximal third of the exopod in *Nitocra taylori* sp. n., but well developed, more elongate and reaching far beyond the middle of the exopod in *Nitocra husmanni*).

### Ecological remarks

The nature of freshwater deprivation in the St Lucia Estuary has resulted in a northward gradient of drought effects. While regions in the south have recently been relatively protected from the drought, due to freshwater input from the Mpate and Mfolozi Rivers through the link canal (Whitfield and Taylor 2009), hypersalinity and low water levels have become increasingly more severe towards the north. This profile has fractionated the system into a variety of different habitats in relatively close proximity.

The first record of *Nitocra taylori* sp. n. in the St Lucia Estuary dates back to 2006, when low densities were collected from Charters Creek, which is situated on the western shore of South Lake. It is possible that this species was present in earlier assessments conducted by Grindley (1976), however, in these assessments, harpacticoid copepods were not identified to species level. The distribution of *Nitocra taylori* sp. n. within the St Lucia Estuary appears to be limited to Catalina Bay and Charters Creek in South Lake and Listers Point in False Bay. No specimens have been recorded from the Mouth or Narrows regions. During closed mouth conditions, False Bay and South Lake are generally characterised by low water levels, high salinities and high turbidities, particularly at Charters Creek and Listers Point. The Mouth and Narrows, on the other hand, are relatively deeper stations with higher water levels and lower salinities.

During this study, *Nitocra taylori* sp. n. individuals were recorded at salinity levels ranging from 9 to 53.7 psu. Many species of *Nitocra* exhibit wide salinity tolerance, since they occur in a variety of different habitats (rock pools, lagoons and sandy beaches), which naturally experience wide salinity fluctuations (Matias-Peralta et al. 2005). In a study by Matias-Peralta et al. (2005), *Nitocra affinis* was capable of tolerating salinity levels of 10 to 35 psu. Although this species was capable of surviving a broad range of salinities, reproductive capacity was hindered at the lower part of the salinity range, while levels of 30 to 35 psu were found to provide the best conditions for development. *Nitocra spinipes* has a slightly wider salinity tolerance, surviving salinity levels ranging from 0.5 to 30 psu (Wulff 1972). In the St Lucia Estuary, *Nitocra taylori* sp. n. was not found at salinity levels lower than 9 psu. It is, therefore possible that the low salinities prevailing in the lower reaches of the estuary are restricting *Nitocra taylori* sp. n. populations to the lakes region. While a large number of individuals were collected at salinities around 100 psu, these individuals were completely transparent, (having not taken up the stain) and resembled discarded exoskeletons. It is most likely, therefore, that they were not alive at the time of collection. Those individuals that did take up the phloxine stain were only recorded at salinity levels up to 53.7 psu.

Matias-Peralta et al. (2005) also found that low light intensities were more favorable for the overall reproduction, population growth and development of *Nitocra affinis*. The high turbidity levels experienced in the lakes of the St Lucia Estuary may, therefore, be favorable to the development of *Nitocra taylori* sp. n. to a certain extent and may also play a role in its distribution within the system. Lastly, water levels in the Mouth and Narrows are deeper than those found in the lakes, which, during closed-mouth conditions, are usually only ~20 cm deep. It is, therefore, possible that shallow water depths are favoured/ required for the survival of this species.

Species belonging to the genus *Nitocra* are also known to inhabit a wide range of sediment types (Boxshall and Halsey 2004). Pillay and Perissinotto (2008) classified the sediment of the St Lucia Estuary in 2005. Average values (±SE) of sediment particle size (phi) indicate that sediment was generally medium to very fine sand at the mouth (3.1 ± 0.5), but was finer at Esengeni (4.08 ± 0.89) where it was classed as very fine sand to coarse silt. Sediments in the South Lake were classed as fine to very fine sand (Charters Creek: 2.48 ± 0.44, Catalina Bay: 2.38 ± 0.48). The finest sediments were recorded at Listers Point in False Bay (6.63 ± 0.86), where they were classed as fine silt. This station, therefore, exhibited the highest silt content of 73.38 ± 6.55 % (Pillay and Perissinotto 2008). Within the St Lucia estuarine lake, *Nitocra taylori* sp. n. individuals were found in both the South Lake and False Bay which have a variable sediment composition ranging from fine silt to fine sands. Similar sediment is found in other areas of the estuarine lake (i.e. Mouth and Narrows), however, no *Nitocra taylori* sp. n. individuals were recorded here. It is, therefore, more likely that a parameter other than sediment size is restricting their distribution.

Whether the main controlling factor is salinity, turbidity, water level, sediment composition, or a combination of all of them, the distribution of this new potentially endemic species is clearly limited to the lake part of the estuary, an area which is most severely affected by the current freshwater deprivation crisis. It appears that this species cannot tolerate salinity levels above 53.7 psu, however, in the current state of the estuary; salinity levels at Charters Creek and Listers Point often exceed this value. Continued freshwater deprivation would, therefore, further limit the distribution range of this species, but may also threaten its survival within the system. Up until now, research within the St Lucia Estuary has been focused at 5 representative stations, however, further investigations are needed in order to document the full extent of the distribution of *Nitocra taylori* sp. n. within the lakes. Additionally experimental studies on the salinity and temperature tolerance limits of this species would aid in the understanding of the physiological factors which affect its survival.

## Supplementary Material

XML Treatment for Nitocra taylori
